# An intervention to improve mental health care for conflict-affected forced migrants in low-resource primary care settings: a WHO MhGAP-based pilot study in Sri Lanka (COM-GAP study)

**DOI:** 10.1186/1745-6215-14-423

**Published:** 2013-12-09

**Authors:** Chesmal Siriwardhana, Anushka Adikari, Tine Van Bortel, Paul McCrone, Athula Sumathipala

**Affiliations:** 1 Institute of Psychiatry, King’s College London, De Crespigny Park, London, SE5 8AF, UK; 2 Institute for Research and Development, Colombo, Sri Lanka; 3 Institute of Public Health, University of Cambridge, Cambridge, UK

**Keywords:** mhGAP, Mental health, Treatment gap, IDP, Sri Lanka, RCT

## Abstract

**Background:**

Inadequacy in mental health care in low and middle income countries has been an important contributor to the rising global burden of disease. The treatment gap is salient in resource-poor settings, especially when providing care for conflict-affected forced migrant populations. Primary care is often the only available service option for the majority of forced migrants, and integration of mental health into primary care is a difficult task. The proposed pilot study aims to explore the feasibility of integrating mental health care into primary care by providing training to primary care practitioners serving displaced populations, in order to improve identification, treatment, and referral of patients with common mental disorders via the World Health Organization Mental Health Gap Action Programme (mhGAP).

**Methods/Design:**

This pilot randomized controlled trial will recruit 86 primary care practitioners (PCP) serving in the Puttalam and Mannar districts of Sri Lanka (with displaced and returning conflict-affected populations). The intervention arm will receive a structured training program based on the mhGAP intervention guide. Primary outcomes will be rates of correct identification, adequate management based on set criteria, and correct referrals of common mental disorders. A qualitative study exploring the attitudes, views, and perspectives of PCP on integrating mental health and primary care will be nested within the pilot study. An economic evaluation will be carried out by gathering service utilization information.

**Discussion:**

In post-conflict Sri Lanka, an important need exists to provide adequate mental health care to conflict-affected internally displaced persons who are returning to their areas of origin after prolonged displacement. The proposed study will act as a local demonstration project, exploring the feasibility of formulating a larger-scale intervention study in the future, and is envisaged to provide information on engaging PCP, and data on training and evaluation including economic costs, patient recruitment, and acceptance and follow-up rates. The study should provide important information on the WHO mhGAP intervention guide to add to the growing evidence base of its implementation.

**Trial registration:**

SLCTR/2013/025.

## Background

Inadequacy in mental health care in low and middle income countries (LMIC) has been an important contributor to the rising global burden of disease [1]. The treatment gap in mental health in LMIC has been well researched, and a strong evidence base is rapidly building through numerous epidemiological and interventional studies in resource-poor settings [2,3]. The importance of incorporating mental health care into existing primary care systems in LMIC has been recognized as a priority by global health policymakers such as the World Health Organization (WHO), global leaders in mental health research, and national governments of LMIC [4-6]. Scaling up of primary care services and task-shifting approaches have been recognized as key concepts in integrating mental health with primary care provision in resource-poor settings [7,8].

Common mental disorders (CMD) are a broad spectrum of conditions, which include depressive and anxiety disorders [9]. They are predominantly present in primary care settings, especially in LMIC [10,11]. However, low detection or non-detection by primary health care providers, increased rates of symptomatic treatment, low referral rates for specialist care, and increased healthcare costs are all associated with CMD in primary care settings in LMIC [10,12]. Lack of adequate training for primary care physicians, lack of adequate human resources (number of physicians), inadequate health systems, and lack of adequate policy attention are seen as the main barriers to effective treatment of CMD through integrating mental health into primary care in LMIC [10,11,13].

In conflict-affected situations, existing resource-limited health systems can be severely affected, limiting any provision of care to populations; this is especially salient for forced migrants in LMIC [14]. Mental health care may be considered a secondary consideration in post-conflict settings, where primary care resources are usually allocated for more immediate health concerns [15,16]. However, there is strong evidence that conflict-driven forced migrants have increased rates of mental disorders including CMD and post-traumatic stress disorder [17,18]. Primary care, however limited, is often the only available service option for the majority of forced migrants, except for those refugees who manage to migrate to more resourceful countries or regions. Integration of mental health into primary care for conflict-affected forced migrants is adifficult task given the political, cultural, economic, and security complications involved. However, there is some evidence that such integration is feasible even in difficult settings, with the potential to improve mental health of affected populations [19].

Although it is recognized that Sri Lanka has a very effective primary care system, the primary care treatment gap for mental health is extensive [20]. Owing to the recently concluded three-decade conflict, the country has several resource-poor regions where there are forced internal migrants, with some having lived in prolonged displacement for over 20 years, and return migration having only recently started. Populations of internally displaced persons (IDP) who experienced prolonged forced migration in Sri Lanka have shown increased rates of CMD (18.8%) compared with national figures, especially of somatoform disorders (14% compared with a national prevalence of 4.2%) and major depression (5.1% compared with a national prevalence of 2.6%), and they lack adequate services of specialist mental health care providers, including psychiatrists [18,20,21]. This is evident among the northern Muslim IDP, displaced as a result of conflict in 1990 in the northern province (mainly Mannar district), who have been living in displacement in the Puttalam district of the north-western province, and are currently returning to areas of origin [18]. Return migration areas in the Mannar district in particular lack adequate healthcare facilities to cater for the influx of returnee populations. For those IDP opting to live in Puttalam, available mental health services are also limited, and the Puttalam district did not have the services of a psychiatrist until 2008. Although the Ministry of Health has taken steps to train a new category of Medical Officers of Mental Health (MOMH) to provide increased mental health care in the absence of specialist psychiatrists, their services are also insufficient to match the existing healthcare needs. Primary care physicians, such as general practitioners, medical officers in government primary care facilities, and medical officers in tertiary care outpatient departments located in the Puttalam or Mannar districts are the front line and often the only available option for mental health care of IDP. However, these physicians do not have the adequate training or skills to identify and treat mental disorders in the busy primary care setting in which they work.

Against this background, the proposed pilot study titled "An intervention to improve mental health care for conflict-affected forced migrants in low-resource primary care settings: a WHO MhGAP-based pilot study in Sri Lanka (COM-GAP)" aims to integrate mental health care into primary care by providing training to primary care practitioners (PCP) serving populations of IDP to improve identification, treatment, and referral of patients with CMD via the WHO Mental Health Gap Action Programme (mhGAP). The mhGAP initiative, introduced in 2008 by the WHO, incorporates evidence-based guidelines on managing mental, neurological, and substance abuse disorders in the primary care setting by non-specialist healthcare workers [7,22]. The mhGAP Intervention Guide (mhGAP IG), introduced in 2010, is a valuable manual that can be used as a resource in training non-specialists in primary mental health care, and is currently in its evaluation and implementation stage in several countries [7,8].

The primary objective of the proposed study is to assess the feasibility of improving/increasing identification, treatment, and referral of CMD by PCP working with a group of displaced and returning forced migrants in a resource-poor, rural, primary care setting via a training program based on the WHO mhGAP IG. This will be carried out by developing a pilot randomized controlled trial (ACT) in the north and north-western provinces of Sri Lanka. The main hypothesis of the study is that training intervention using mhGAP IG will increase rates of correct identification, treatment, and specialist referral rates of the PCP. It is envisaged that training PCP to efficiently identify, treat, and refer patients with CMD will reduce the acute and long-term disease burden, reduce service-provision costs, and provide effective/accessible mental health care to vulnerable/traumatized IDP. As South Asia faces continuous problems relating to mass forced migration, this study may provide important evidence on reducing the treatment gap for vulnerable populations in the region, with a high potential for scaling up in other resource-poor settings. This is the first study of its kind to be undertaken in Sri Lanka, and is planned as a local demonstration study to aid a future larger-scale intervention.

## Methods/design

### Study design and setting

The study will be carried out in primary care settings (outpatient departments of government hospitals, government rural hospitals, government central dispensaries, and private medical clinics) in the Puttalam and Mannar districts of the north and north-western provinces of Sri Lanka, where conflict-affected, long-term displaced/returning migrants are located. It will be conducted as a feasibility study in the form of a pilot RCT.

### Participants

Two groups of participants will be recruited. The main participant group will be PCP working in government health services or private practices in the primary care institutions listed above within the geographical areas of the Puttalam and Mannar districts. The secondary participant group will be patients diagnosed with CMD (depression, anxiety, somatoform disorder/medically unexplained symptoms, or suicidal ideation) by the recruited PCP during the study period. PCP will be approached at the primary care institutions (government and/or private) by the principal investigator (PI) and the research team. They will also be approached (as applicable) through the Sri Lanka College of General Practitioners and other relevant organizations. Appropriate permission has been sought and obtained from the Ministry of Health and Provincial Ministries of Health.

### Inclusion and exclusion criteria

To be included, PCP will need to: have 5 or more years of experience, have full registration with the Sri Lankan Medical Council, and be providing a minimum of 4 hours/day of primary care to IDP populations. PCP will be excluded if: they are psychiatrists, have secondary mental health training, less than 5 years of service, and work less than 4 hours/day with IDP populations.

To be included, patients will need to: belong to a specific IDP groups, and be adult (18 years or older). Patients aged under 18 years will be excluded, as the intervention training focuses on adult population. Patients diagnosed with serious mental disorders will also be excluded as the intervention training focus is on CMD.

### Sampling

It must be highlighted that this is purely a feasibility study. Therefore, as a pilot RCT, 43 PCP for each arm (total of 86) are estimated to be a sufficient sample size, including drop-out allowance [23,24]. Specific global evidence required for sample size calculation through standard methods is not available. Sample size calculations for patients are not required, as they will not be included in the trial, and no outcomes will be measured for this group (more details explained in the next section).

### Intervention

The training intervention will be based on the mhGAP IG developed by the WHO. Only relevant modules (depression, medically unexplained symptoms, alcohol abuse, and suicide) will be used for the training. The training will be based on a complete training guide and material provided by the WHO, which contains the necessary course material for trainees and preparatory and guidance materials for trainers. It will be conducted by the PI (who has been trained by the Royal College of Psychiatrists affiliated mhGAP training programme, UK), mhGAP IG experts, and another clinically experienced psychiatrist. Training programs will be conducted as a full-day course for five consecutive days, as originally designed by the WHO. The training intervention will be given to 43 (approximately half) of the recruited PCP in the intervention arm, randomized by appropriate techniques. Participants in the control arm will be given a similar training in mhGAP after the completion of the study.

Training will take place after an initial 3 month monitoring period of the whole sample from the date of recruitment (90 day monitoring period for each participant from the date of recruitment). During this period, all PCP are required to inform the study coordinator immediately on diagnosing a primary care patient with CMD. The PCP is required to obtain written informed consent from each patient if they agree to be contacted by the research team, prior to informing the coordinator. All PCP will be given initial training on ethics and informed consent procedures prior to commencing the study.

After the 5 day training period of the intervention group, both arms will be monitored again for a 3 month period. Those in the intervention arm will be required to use the mhGAP guide to diagnose, treat, and refer patients with possible mental health problems whom they encounter in their respective primary care settings. The control arm will be requested to continue their existing diagnostic and treatment practices. Participants in both arms will be required to communicate any CMD diagnoses to the study coordinator immediately.

During the first and second 3-month monitoring periods, the study coordinator will gather a list of patients diagnosed by the participant PCP (from both intervention and control arms) on a daily and weekly basis. These patients will then be approached individually by the study coordinator, who will explain the purpose of the study. The coordinator will provide an information leaflet and obtain secondary written consent from each patient. Consenting patients will then be directed to the study psychiatrist, who will reassess them according to mhGAP-based criteria for concurrence with the initial PCP diagnosis. Unless absolutely necessary, the psychiatrist will not modify or change the treatment or management outlined by the PCP. For those patients categorized as referrals to specialist care, their treatment and management will be provided by the study psychiatrist. However, referral patients will be completely free to decide if they want to be treated by the study psychiatrist or to seek care from other government or private specialist psychiatrists. All patients consenting to be involved in the study will be compensated for travelling costs or provided with travel if they prefer. No other compensation will be offered. For COM-GAP study phases according to CONSORT guidelines, please see the Additional file 1.

### Randomization allocation

All PCP recruited to the study will be randomized into the intervention and control arms by the study statistician using standard randomization techniques. It is not necessary to randomized the the patients.

### Allocation concealment

It will not be possible to mask the study team to the status of the PCP, as they will be aware of the PCP participants in the training intervention arm. The study psychiatrist will be blinded/masked to the randomization status of the PCP, and therefore be unbiased with regard to the patients originating from each PCP (that is, whether a patient was diagnosed by a trained or untrained PCP). The study psychiatrist will therefore not be involved in the training program. In addition, patients will also be blinded to the PCP randomization status.

### Minimization of contamination

The PCP will be working in close, possibly overlapping geographical locations, thus there is a theoretical possibility of contamination by recruits having personal knowledge of each other. Additionally, there is a possibility of PCP in different study groups exchanging information received in the training. While we acknowledge that such interaction may potentially lead to contamination, it should be noted that this will be extremely difficult to prevent, and is not likely have a significant influence on the outcome. Because the training provided during the study is very thorough, and the study group participants are required to carry out intensive tasks, contamination through exchanges of information in informal discussions is not anticipated. However, as a preventive action, both the study and control group PCPs will be requested to refrain from seeking study-related information from each other for the duration of the intervention. At all possible times during recruitment, training, and monitoring, steps will be taken to observe potential contamination. At the end of the study, information will be gathered separately from both groups in order to gain an insight into potential contamination. However, it must be mentioned that, as this is a feasibility pilot study, minor contamination may not affect the outcomes to a large degree.

### Measurements

A sociodemographic questionnaire will be administered to all PCP, and this will include a section on medical education and experience at the point of recruitment. At baseline, PCP will also be given knowledge assessment questionnaires on each module included in the intervention training, to measure their existing knowledge. This will be repeated for the intervention arm during the training program at the start of each module. The knowledge-assessment questions to be used have been designed by the WHO to match the mhGAP training package. The same measure will be given to the control arm (without the training) at the same timepoint as the intervention arm. Post-training knowledge assessments (designed by the WHO) will be administered to the intervention arm at the end of the 5 day training program, and to the control arm at the same time. For both arms, knowledge assessment will be repeated at the end of the study period.

All patients who consent to participate will be given a sociodemographic questionnaire. At the point of recruitment, they will also be given an appropriate language version of the Patient Health Questionnaire (PHQ) as a screening measure [25]. The PHQ has been translated and used in both Sinhala and Tamil languages in the Sri Lankan IDP population [18]. The final instrument administered to patients will be the Sri Lankan version of the Client Service Receipt Inventory (CSRI) [26-28] to record health service utilization, including time lost from work. Both PHQ and CSRI will be administered at the point of recruitment and after the 3 month follow-up period.

### Primary outcomes and statistical analysis

Referral rates before and after the intervention will be measured for all PCP. Rates of study psychiatrist-verified diagnoses will be measured. Quality of adherence to mhGAP guidelines for treatment and management will be assessed for the intervention arm. Knowledge levels at the three test points will be measured for all PCP. For PCP in the intervention arm, knowledge levels will be assessed during each day of the training program. Overall comparisons will be made between the intervention and control arm rates for referral, correct diagnosis, and knowledge increase. Secondary outcomes include concurrence between the clinical diagnosis and the screening instrument and basic economic evaluation (described later).

No interim analysis will be carried out, and analysis will commence only at the end of the full study period. Because this is a pilot study, the analysis will be focused on primary feasibility, and therefore will primarily be descriptive in nature, with estimations of confidence intervals as appropriate [24]. As power calculations will not be carried out, it would not be appropriate to conduct analyses aimed at testing hypotheses [24]. The main analysis will be based on difference in outcomes of intervention and control arms using two-way analysis of variance (ANOVA). Appropriate consideration will be given to potential confounders. Results will be used to formulate sample size calculation for a larger intervention.

### Data management and trial procedures

Double data entry will be conducted by a team of data entry operators blinded to details of allocation. A statistician and a data manager will supervise the data entry process. The PI will be responsible for the overall management and supervision of the study. The PI has previous experience as a clinical trial monitor for an international clinical trial, and has been fully trained in Good Clinical Practice (GCP) guidelines. He is trained to deliver the mhGAP course by the Royal College of Psychiatrists in the UK. He has also conducted two large-scale epidemiological surveys of the IDP population in the Puttalam and Mannar districts, and has built a high level of rapport with the communities. The study will be coordinated by a medically trained researcher with previous coordination experience. The coordinator will be responsible for liaising with PCP, recruiting PCP and patients, and coordinating the patient referrals to the study psychiatrist. The study psychiatrist has experience in working with clinical populations in Sri Lanka for a number of years. The overall supervision of the study will be performed by a highly experienced consultant psychiatrist with extensive research and clinical experience in both Sri Lanka and the UK.

### Qualitative component

A small-sample qualitative study nested within the pilot RCT will be carried out to explore the attitudes and perspectives of PCP on the importance of integrating mental health into primary care. The qualitative component will be carried out prior to the main study in order to provide feedback for the training intervention. Participants (approximately 20) will be recruited from within the main study PCP cohort. Informed written consent will be obtained separately for the qualitative component. An in-depth individual interview based on a topic guide will be conducted by a trained qualitative researcher to elicit views, perceptions, and attitudes of PCP towards mental health, importance of providing mental health care, integration of mental health into primary care settings, perceived barriers and requirements for such initiatives, and other related topics. All interviews will be conducted in English, at a location preferred and convenient to participants, and audio-recorded with consent. Maximum confidentiality will be assured throughout the process of recruitment, interview, and subsequent analysis. Audio recordings will be transcribed verbatim, and subsequently analyzed using the thematic analysis model.

### Economic evaluation

The study will calculate the costs incurred in training the PCP in mental health care, and the extra time they may spend with patients. These costs will be linked to the primary outcome measure in order to determine the extra cost incurred to detect one extra patient. Other service use data (primary care, secondary care, social care, input from families) will be recorded through the CSRI for patients identified with CMDs, and costs will be calculated using unit costs specific to Sri Lanka. These costs will be added to the intervention costs, and will be compared with information from other more resource-rich populations to understand the economic implications of service provision to vulnerable populations. The number of patients receiving treatment will be identified, and the disability adjusted life years (DALYs) calculated. The cost per DALY avoided will subsequently be calculated. Research findings have shown that higher rates of CMD are associated with unemployment in IDP populations in the region, and economic evaluations of mental health care interventions provide important information for IDP management and service provision [18].

### Ethical issues

Ethics approval has been obtained from the Ethics Review Committee, Faculty of Medical Sciences, University of Sri Jayewardenepura, Sri Lanka. Informed written consent will be obtained from eligible PCP after the initial approach, and an information leaflet will be provided to them. An appropriate length of time (minimum 1 day) will be allowed for decision-making. If the PCP is interested, the study coordinator will obtain their written consent. Each patient diagnosed by individual PCP during study period will be asked twice for consent. In first instance, the PCP will explain the study and obtain consent from the patient for the study team to approach the patient. The patient will be told that the treatment will strictly not depend on their decision to participate. PCP will be trained in ethical aspects of informed consent, and will be strongly encouraged to prevent undue inducement or coercion. However, given the doctor-patient relationship in Sri Lanka, this is extremely difficult to prevent. The study team will make regular spot-checks with PCP to ensure that systematic coercion is not present. If a patient consents to be approached by the study team, then the study coordinator will approach the patient and re-explain the study with an information leaflet. Again, after an appropriate time gap, the patient will be asked to decide to participate. If they consent, the study coordinator will make subsequent arrangements for the patient to have a clinical interview with the study psychiatrist. From the moment of recruitment, all patients will be anonymized to the study team, and data will be entered using a numbered coding system. Both PCP and patients will be explicitly informed of the feasibility nature of the pilot study during the recruitment process. Both PCP and patients will be able to withdraw at any time during the study without presenting a reason. Maximum efforts will be made to ensure data protection and confidentiality. If the study psychiatrist discovers significant medical errors in the treatment and management by PCP, they will be individually approached and advised to rectify the errors. As mentioned above, referral patients have the choice to refuse the services of the study psychiatrist and seek specialist care elsewhere. If any serious mental disorders are diagnosed by the PCP during the study period, those patients will be advised to seek immediate specialist advice and refer to specialist care. The study team will provide full support in such situations, both to the PCP and the patient, as required. Information leaflets and consent forms for PCP will be in English, and for patients they will also be available in Sinhala and Tamil.

### Ethics approval

The study has been approved by the Ethics Review Committee, Faculty of Medical Sciences, University of Sri Jayewardenepura, Sri Lanka.

## Discussion

The main purpose of this feasibility study is to integrate mental health into primary health care by training PCP to improve identification, management, and referral of CMD for conflict-affected forced migrants. In post-conflict Sri Lanka, an important need exists to provide adequate mental health care to conflict-affected IDP returning to their area of origin after prolonged displacement. The proposed study will act as a local demonstration project exploring the feasibility of formulating a larger-scale intervention study in the future, and is envisaged to provide information on PCP engagement, training and evaluation, and rates of patient recruitment, acceptance, and follow-up. It is also planned to measure the views, attitudes, and perceptions of PCP on integrating mental health in to primary care. The economic evaluation is aimed at collating information about the costs involved in obtaining and providing mental health care in resource-poor primary care settings, which will aid policy decisions on management of IDP and other vulnerable populations. The study stands to provide important information on the WHO mhGAP intervention guide to add to the growing evidence base of its implementation, which will be helpful to tailor it to training primary care workers in other global conflict-affected populations and humanitarian contexts.

## Trial status

Commenced.

## Abbreviations

An intervention to improve mental health care for conflict-affected forced migrants in low-resource primary care settings: A WHO MhGAP-based pilot study in Sri Lanka (COM-GAP); ANOVA: Analysis of variance; CONSORT: Consolidated Standards of Reporting Trials; CSRI: Client service receipt inventory; DALY: Disability adjusted life year; GCP: Good clinical practice; IDP: Internally displaced people; PCP: Primary care practitioner; PHQ: Patient health questionnaire; mhGAP IG: Mental health gap action programme intervention guide; WHO: World Health Organization.

## Competing interests

The authors declare no competing interests.

## Authors’ contributions

CS conceived the study, and wrote the study protocol and manuscript. TVB, PM, and AS provided critical design input, and reviewed the manuscript. AA was involved in developing the protocol. All authors read and approved the final version of the manuscript.

## Supplementary Material

Additional file 1 Consolidated Standards of Reporting Trials (CONSORT) flow diagram for COM-GAP study phases. Click here for file
