# Supplementary material for: An intervention to improve mental health care for conflict-affected forced migrants in low-resource primary care settings: a WHO MhGAP-based pilot study in Sri Lanka (COM-GAP study)
Source: Trials. 2013 Dec 9;14:423. doi: 10.1186/1745-6215-14-423 (PMC3906999; doi:10.1186/1745-6215-14-423)
Supplement: Additional file 1 — Consolidated Standards of Reporting Trials (CONSORT) flow diagram for COM-GAP study phases. [file 1745-6215-14-423-S1.doc]

**Additional file 1**

**CONSORT Flow Diagram for COM-GAP study phases**

Assessed for eligibility (n=x)

Excluded (n=x )

♦  Not meeting inclusion criteria (n=x)

♦  Declined to participate (n=x)

♦  Other reasons (n=x)

Lost to follow-up (give reasons) (n=x)

Discontinued intervention (give reasons) (n=x)

Lost to follow-up (give reasons) (n=x)

Discontinued intervention (give reasons) (n=x)

**Phase 2 - Allocation & training intervention**

**Phase 4 –Follow-up and monitoring period for 3 months and analysis**

**Phase 3 – WHO mhGAP IG**

5 day training programme for the intervention arm

**Phase 1 – Enrollment & monitoring period for 3 months**

**Pre trial phase – recruitment of primary care practitioners (PCP)**

**Phase 3 – recruitment of patients and clinical validation**

Analysed (n=x)

Allocated to intervention (n=43)

Not allocated to intervention (n=43)

Analysed (n=x)

Randomized (n=apprx.86)
